# Visualization and quantification of dynamic STAT3 homodimerization in living cells using homoFluoppi

**DOI:** 10.1038/s41598-018-20234-2

**Published:** 2018-02-05

**Authors:** Yusuke Okada, Taku Watanabe, Toru Shoji, Kyoko Taguchi, Naohisa Ogo, Akira Asai

**Affiliations:** 1 Centre for Drug Discovery, Graduate School of Pharmaceutical Science, University of Shizuoka, Suruga-ku, Shizuoka, Shizuoka, Japan; 2 Discovery Technology Laboratories, Sohyaku, Innovative Research Division, Mitsubishi Tanabe Pharma Corporation, Kawagishi, Toda-shi, Saitama, Japan; 3 Ina Laboratory, Medical & Biological Laboratories Co., Ltd., Ina, Nagano, Japan

## Abstract

Dimerization in signal transduction is a dynamically regulated process and a key regulatory mechanism. Signal transducer and activator of transcription 3 (STAT3) dimerizes after tyrosine phosphorylation upon cytokine stimulation. Because only the STAT3 dimer possesses the trans-activation activity, dimerization is an indispensable process for cytokine signaling. Here we report the detection of dynamic STAT3 dimerization in living cells using the homoFluoppi system. This method allowed us to validate the presence of an intact Src homology 2 domain and STAT3 Tyr705 phosphorylation, which facilitate puncta formation and homodimerization. Puncta formation was reversible, as determined by a decreased punctate signal after washout of oncostatin M. We analyzed STAT3 mutants, which have been reported in patients with hyper IgE syndrome and inflammatory hepatocellular adenoma (IHCA). Analysis of the IHCA mutants using homoFluoppi revealed constitutive activity independent of cytokine stimulation and novel insight into kinetics of dimer dissociation process. Next, we used homoFluoppi to screen for inhibitors of STAT3 dimerization, and identified 3,4-methylenedioxy-β-nitrostyrene as a novel inhibitor. The results of this study show that homoFluoppi is a useful research tool for the analysis of proteins like STAT3 that dynamically dimerize, and is applicable for the screening of dimerization modulators.

## Introduction

The dimerization of many proteins such as transcription factors or G protein-coupled receptors is an important regulatory mechanism governing cell function. For example, heterodimerization is a mechanism that gives rise to functional diversity. Homodimerization gives rise to unique functions that monomers do not have^1^. Therefore, protein dimerization seems to be an essential mechanism underlying the diverse functions of a limited number of proteins. More importantly, dimerization is a decision point in signal transduction, so dimerization events must be tightly regulated^1^. Excessive and prolonged dimer formation causes problems in cell signaling, which can lead to disease. Thus, analyzing the relationship between mutations in the dimerization motifs of signal transduction molecules and pathogenesis may result in novel therapeutic strategies. Because there are some known causal relationships between aberrant protein dimerization and pathogenesis^2^, investigations of inhibitors or modulators of protein dimerization have garnered attention as potential therapeutic agents^3,4^.

The signal transducer and activator of transcription (STAT) family of transcription factors are proteins that transduce signals upon dimerization^5–7^. The STAT family comprises seven members (STAT-1, -2, -3, -4, -5a, 5b, -6), all of which induce the transcriptional activation of many target genes. STAT proteins are phosphorylated at conserved tyrosine residues by Janus kinases (JAKs) upon receptor binding of cytokines like interleukin 6 (IL-6) or growth factors such as epidermal growth factor (EGF). Each STAT protein has a Src homology 2 (SH2) domain that specifically recognizes phosphorylated tyrosine residues^8^, leading to homodimerization or heterodimerization followed by STAT translocation into the nucleus. Translocated STAT dimers induce the transcription of target genes. Activation and inactivation of STAT signaling must be tightly regulated, because dysregulation of STAT signaling has been implicated in some pathological conditions^9^. For example, dysregulated activation of STAT3 has been observed in many types of cancer cells, so STAT3 is expected to be the effective target for anticancer therapy^10–12^.

To date, several approaches have been taken to modulate dysregulated STAT3 activation. For example, STAT3 is activated by JAK-mediated phosphorylation, so inhibition of upstream JAK kinases is a potential way to inhibit STAT3 activation. JAK inhibitors have been tested in clinical trials for the treatment of human tumors^13^. However, STAT3 is also phosphorylated and activated by other kinases, and even some STAT3 mutations are activating^14^. Therefore, approaches for inhibiting STAT3 homodimerization and modulating its activity have garnered much attention^10–12^, as these compounds are expected to have anticancer activity.

Despite the importance of modulating dimer formation as a potential therapeutic strategy, research methods for analyzing the dynamic dimerization process in normal and disease states, and screening methods for identifying dimerization modulators, especially in living cells, are limited. The conformation of proteins is influenced by changes in the environment such as pH and ionic strength, and also by macromolecular crowding^15,16^. Some recombinant proteins such as STAT5 are difficult to express as a full-length soluble protein^17^. In addition, protein dimerization is regulated by proteins that interact transiently or sequentially. Therefore, a method that is suitable for screening in living cells is especially desired.

Immunoprecipitation (IP), fluorescence resonance energy transfer (FRET)^18,19^, and bioluminescence resonance energy transfer (BRET)^20^ are methods that have been used to detect STAT3 dimerization in living cells. However, it is difficult to detect the dynamic dimerization process by IP assays, as they do not have sufficient throughput. In addition, it is not easy to quantify dimerization by IPs. FRET and BRET assays are difficult and time-consuming for optimizing constructs such as the length of linker sequences. Another method that could be used to detect STAT3 dimerization is the split-green fluorescent protein-based biomolecular fluorescence complementation (BiFC) assay, which is based on formation of a fluorescent complex when two proteins fused to non-fluorescent fragments of a fluorescent protein interact with each other. However, the complex that forms is irreversible, so once the dimer forms it is difficult to dissociate^21,22^. Therefore, to overcome these shortcomings, a novel method that enables the detection of homodimerization in living cells easily, quantitatively, and reversibly is strongly desired.

Fluorescent protein-protein interaction visualization (Fluoppi) is a system that allows the detection of protein-protein interactions (PPIs) reversibly and quantitatively in living cells^23^. In this system, two tags, a Phox and Bem1p tag (PB1-tag) and a tetrameric Azami-Green fluorescent protein tag (AG-tag), are fused to the protein of interest. When the tagged proteins interact, they are immediately crosslinked to form condensed phase separated droplets. The fluorescent protein tags in this droplet are detected as fluorescent puncta in a reversible manner. Although the Fluoppi system is useful for detecting PPIs in living cells^24–26^, it was recently extended to allow visualization of protein homodimerization by development of a single fusion construct containing PB1 and mAG1 (homoFluoppi) that is fused to a protein of interest^23^.

Therefore, in this study, we applied homoFluoppi to detect STAT3 homodimerization in living cells. We validated this novel method by analyzing the regulatory mechanisms underlying STAT3 homodimerization. Then we used homoFluoppi to analyze the effects of STAT3 mutations related to disease. Finally, we conducted high throughput screening (HTS) for inhibitors of STAT3 homodimerization in living cells, and identified 3,4-methylenedioxy-β-nitrostyrene (MNS) as a new dimerization inhibitor.

## Results

### Selection of the optimal STAT3 fusion protein for detection of dimerization in living cells

**Figure 1:**
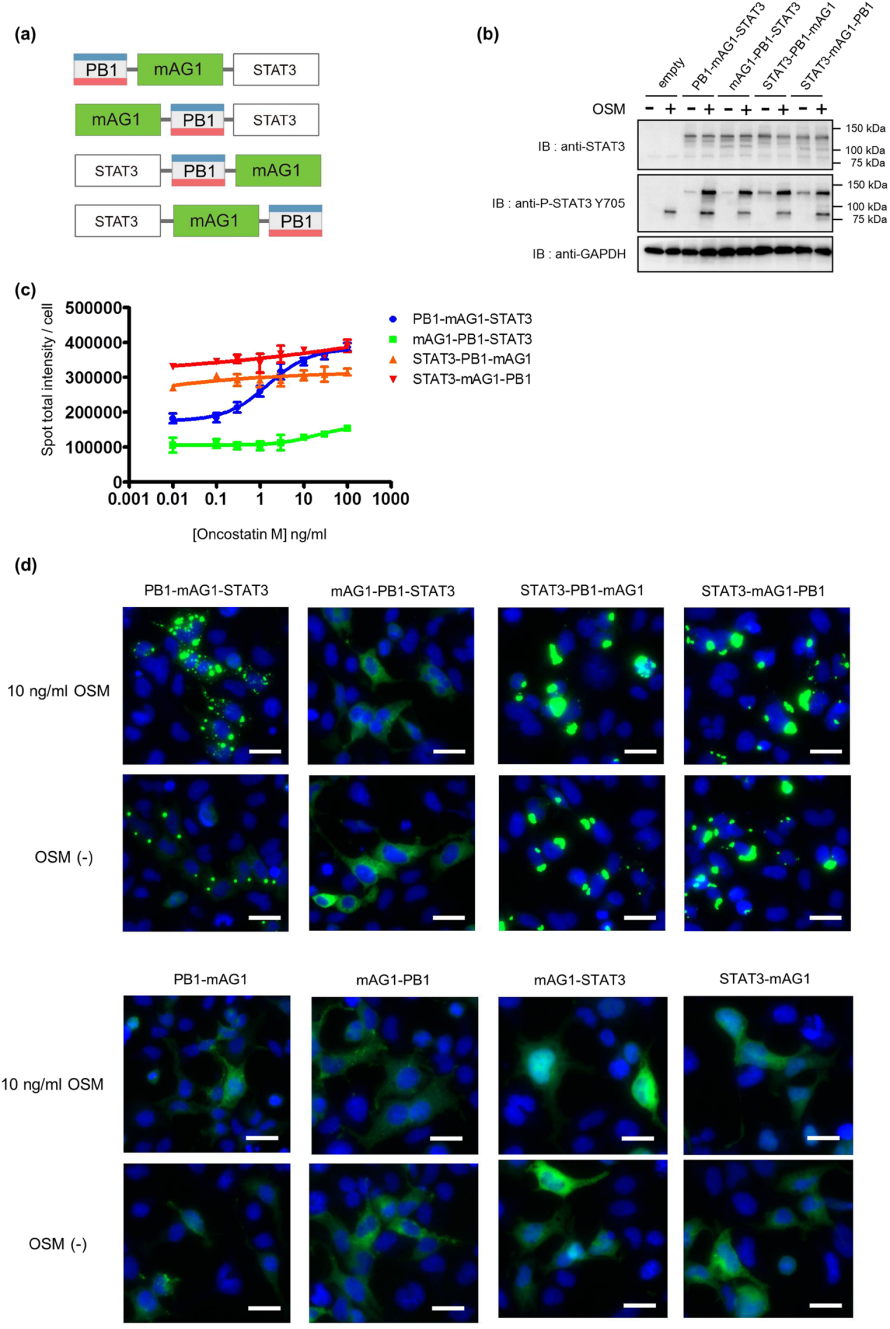
Selection of the optimal construct for detection of STAT3 dimerization in living cells. (**a**) Schematic diagram of four potential homoFluoppi constructs. Red and blue in PB1 indicate acidic and basic regions, respectively, used for self-association in equilibrium. (**b**) Western blot analysis of cells transiently expressing the indicated STAT3 fused to the homoFluoppi tag. GAPDH was used as a loading control. OSM was treated for 30 min. The full-length blots were cropped to improve clarity. Full-length blots are presented in Supplementary Figures 5. (**c**) Quantification of homoFluoppi fluorescent puncta. Fluorescent punctate images were obtained in ArrayScan XTi and analyzed with manufacture’s software to quantification. Dose (OSM)-response (fluorescent punctate intensity per cell) curve for cells expressing indicated homoFluoppi constructs. Cells were treated with OSM for 1 h. Fluorescent punctate intensity per cell was obtained by dividing the total mAG1 (green) fluorescent puncta by the total number of cell (nuclei) which had fluorescence in each field of view. Each point represents the mean of four replicates, and the error bars represent the standard deviation from the mean. (**d**) Fluorescent images of cells expressing the indicated homoFluoppi construct with (top) or without (bottom) OSM. Images of mAG1 (green) and Hoechst33342-stained nuclei (blue) were merged. Scale bars, 25 μm.

In the homoFluoppi system, when proteins fused to PB1/mAG1 tags interact and form homodimers, the homodimers are observed as fluorescent puncta^23^. There are four ways that STAT3 can be tagged with the two Fluoppi tags (Fig. 1a). To select the best tag position for detecting STAT3 in living cells, we constructed four different STAT3 fusion vectors and transfected them into human embryonic kidney 293 (HEK293) cells, as these cells have little endogenous STAT3 expression. STAT3 forms homodimers in response to stimulation by cytokines such as IL-6, interferon alpha (IFN-α), and oncostatin M (OSM), so we treated the transfected cells with OSM. First, we confirmed expression of the tagged STAT3 proteins in the transfected cells (Fig. 1b). We were able to differentiate between endogenous STAT3 (~88 kDa) and exogenous Fluoppi (PB1 and mAG1) tagged STAT3 (~130 kDa) by western blotting because of their different molecular weights. We also confirmed the phosphorylation of tyrosine 705 (Y705) in response to OSM stimulation of both endogenous and exogenous STAT3. To select the best fusion protein to use for STAT3 detection, we observed and quantified the punctate signal (fluorescent punctate intensity per cell) according to time and dose after OSM stimulation using the Spot Detector Bioapplication protocol of ArrayScan. We observed a small increase in punctate signal in cells transfected with mAG1-PB1-STAT3, STAT3-PB1-mAG1, and STAT3-mAG1-PB1 (Fig. 1c,d, Supplementary Fig. 1a,b). However, we visualized a large increase in punctate signal in cells transfected with PB1-mAG1-STAT3 (Fig. 1c,d, Supplementary Fig. 1c). In addition, cells expressing this construct were more sensitive to low concentrations of OSM than cells expressing the other three constructs (Fig. 1c). Cells expressing mAG1-STAT3, STAT3-mAG1, mAG1-PB1, or PB1-mAG1 control constructs did not form puncta in response to OSM (Supplementary Fig. 1d). We also confirmed that PB1-mAG1-STAT3 formed puncta in response to IL-6 or IFN-α stimulation, although lower signal intensity than OSM (Supplementary Fig. 1e). The punctate signals induced by those cytokines were correlated with the results in STAT3 reporter gene assay (data not shown). Consequently, we determined PB1-mAG1-STAT3 to be the best construct for detecting STAT3 dimerization in living cells. Next, we observed puncta in cells expressing PB1-mAG1-STAT3 to determine its localization using an ArrayScan microscope. Fluorescent images showed that PB1-mAG1-STAT3 was mainly distributed in a diffuse pattern in the cytoplasm without OSM stimulation, but formed puncta mainly in the cytoplasm with OSM stimulation (Supplementary Fig. 2a). To determine whether PB1-mAG1-STAT3 translocates to the nucleus and induces transcription activation, we analyzed its transactivation activity using a STAT3-dependent reporter assay. As a result, cells expressing PB1-mAG1-STAT3 did not show transactivation activity than cells expressing only endogenous STAT3, where cells overexpressing untagged STAT3 increased the luciferase signal (Supplementary Fig. 2b).

### Analysis of PB1-mAG1-STAT3 dynamic (reversible) homodimerization in living cells

#### Analysis of STAT3 activation using homoFluoppi

**Figure 2:**
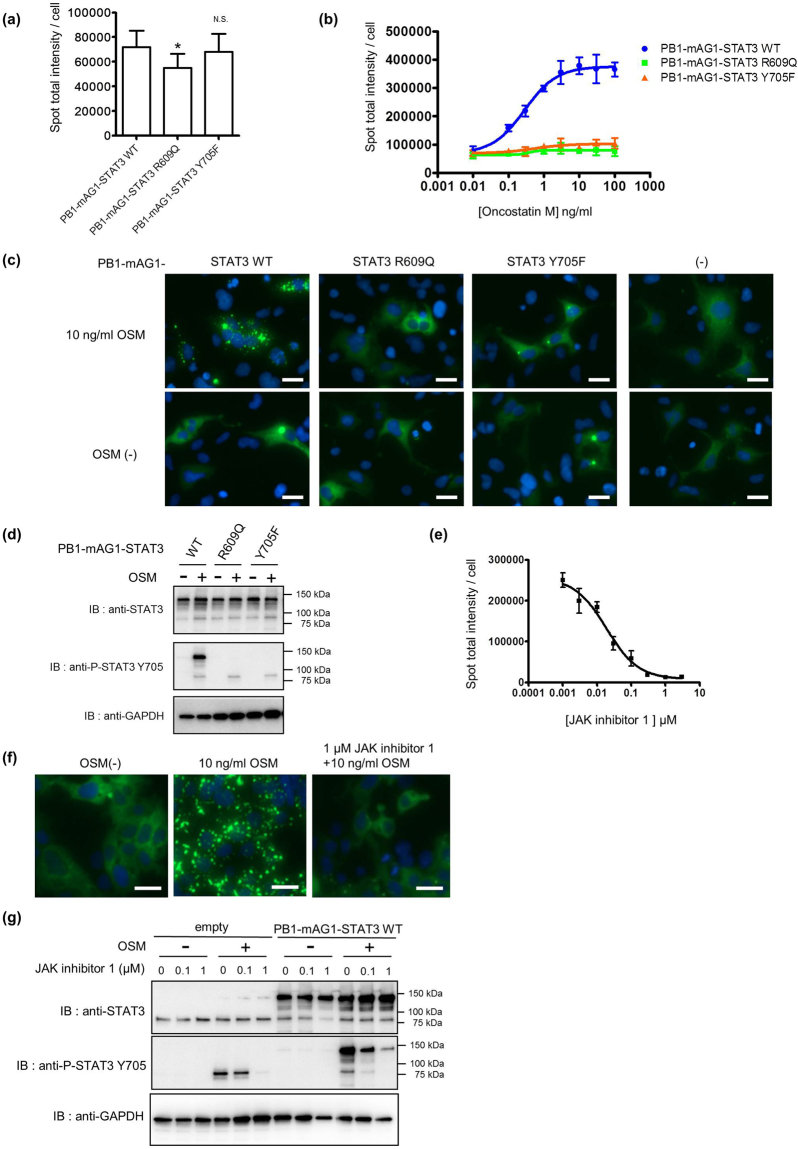
Analysis of STAT3 activation using homoFluoppi. (**a**) Fluorescent punctate intensity of cells expressing the indicated homoFluoppi constructs without OSM. Each data point represents the mean of eight replicates, and the error bars represent the standard deviation from the mean. Statistical significance was examined in the Student’s *t-*test with Bonferroni correction (*p < 0.05 versus WT; N.S., not significant). (**b**) Dose (OSM)-response (fluorescent punctate intensity per cell) curve for cells expressing indicated STAT3 homoFluoppi constructs. OSM treatment for 1 h. Fluorescent punctate intensity per cell was obtained by dividing the total mAG1 (green) fluorescent punctate intensity by the total number of cell (nuclei), which had fluorescence in each field of view. Each point represents the mean of four replicates, and the error bars represent the standard deviation from the mean. (**c**) Fluorescent images of cells expressing the indicated homoFluppi construct with (top) or without (bottom) OSM. Images of mAG1 (green) and Hoechst33342-stained nuclei (blue) were merged. Scale bars, 25 μm. (**d**) Western blot analysis of cells transiently expressing the indicated STAT3 homoFluoppi constructs. OSM treatment for 30 min. Full-length blots are presented in Supplementary Figures 5. (**e**) Dose (JAK inhibitor 1)-response (fluorescent punctate intensity per cell with or without 3 ng/mL OSM) curve for cells stably expressing PB1-mAG1-STAT3. JAK inhibitor 1 was added 30 min before OSM stimulation. OSM treatment for 1 h. Each point represents the mean of four replicates, and the error bars represent the standard deviation from the mean. (**f**) Fluorescent images of cells stably expressing PB1-mAG1-STAT3 with (middle) or without (left) OSM. JAK inhibitor 1 (right) treatment was 30 min before OSM stimulation. Images of mAG1 (green) and Hoechst33342-stained nuclei (blue) were merged. Scale bars, 25 μm. (**g)** Western blot analysis of HEK 293 cells (left) or cells stably expressing PB1-mAG1-STAT3 (right). Cells were treated with the indicated concentration of JAK inhibitor 1 for 30 min, followed by treatment with or without 3 ng/mL OSM for 30 min. Full-length blots are presented in Supplementary Figure 5.

Binding of cytokines such as OSM or IL-6 activates downstream kinases like JAKs, resulting in phosphorylation of the glycoprotein 130 (gp130) receptor. STAT3 is recruited to phosphorylated gp130 via its SH2 domain, after which it is phosphorylated at a conserved Y705 residue by JAKs^7,27–29^, leading to its homodimerization^5^. Thus, STAT3 homodimerization is facilitated by Y705 phosphorylation^30^. Previous reports have shown that STAT3 forms a latent homodimer or oligomer independent of cytokine stimulation or tyrosine phosphorylation^31,32^. According to FRET analysis, the STAT3 R609Q mutant, which is located in the SH2 domain, does not form a latent dimer^18^. In addition, the STAT3 Y705F mutant does not form a homodimer in response to cytokine stimulation^18^. Therefore, we used the homoFluoppi system to determine how these two STAT3 mutants would affect punctate formation. There was a lower punctate signal in the presence of PB1-mAG1-STAT3-R609Q compared with wild-type (WT) STAT3 in the unstimulated condition (Fig. 2a), and the signal did not increase with OSM stimulation (Fig. 2b,c). Western blot analysis confirmed that the PB1-mAG1-STAT3-R609Q mutant had been successfully transfected into cells, but phosphorylation at Y705 after OSM stimulation did not occur (Fig. 2d). As expected by the previous report, punctate signals in cells expressing PB1-mAG1-STAT3-Y705F mutants did not change with OSM stimulation (Fig. 2b,c)^18^. To determine if STAT3-mediated transcription occurred in the transfected HEK293 cells, we conducted a reporter assay with the WT and STAT3 mutants. STAT3 WT showed dose-dependent transcriptional activation upon OSM stimulation, and the STAT3 R609Q and Y705F mutants did not show OSM-dependent transcription of the STAT3 reporter gene (Supplementary Fig. 3a). Next, we analyzed whether punctate signals observed by OSM stimulation were affected by inhibition of upstream JAK activity using JAK inhibitor 1 (2-(1,1-dimethylethyl)−9-fluoro-3,6-dihydro-7H-benz[h]-imidaz[4,5-f]isoquinolin-7-one), which was known to be the ATP competitive pan-JAKs inhibitor^33^. Addition of the JAK inhibitor 1 decreased STAT3 punctate signals that had been increased by OSM in a dose-dependent manner (Fig. 2e,f), and also decreased phosphorylation of endogenous and exogenous STAT3 (Fig. 2g). Together, these data suggest that punctate signals formed by PB1-mAG1-STAT3 are facilitated by tyrosine phosphorylation and an intact SH2 domain. Accordingly, we conclude that the observed formation of STAT3 homodimerization using homoFluoppi was in response to physiological activation regulatory mechanisms.

#### Analysis of STAT3 inactivation using homoFluoppi

**Figure 3:**
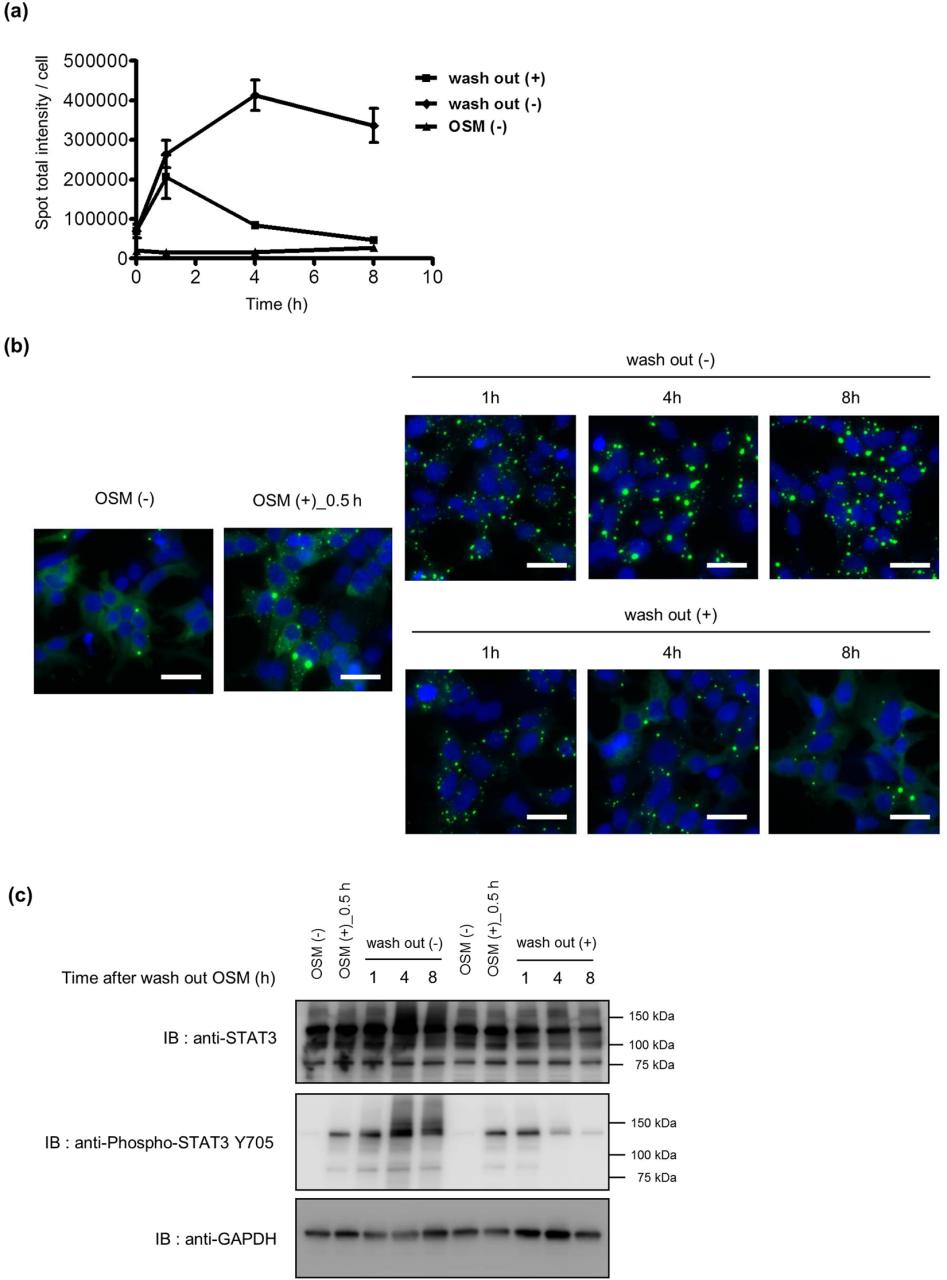
Analysis of STAT3 inactivation using homoFluoppi. (**a**) Cells stably expressing PB1-mAG1-STAT3 were treated with 1 ng/mL OSM for 30 min. Then OSM was washed out and cells were incubated in medium without OSM for the indicated time. As a control experiment, cells without OSM stimulation and cells without OSM washing are shown. Each point represents the mean of four replicates, and the error bars represent the standard deviation from the mean. (**b**) Representative fluorescent images of cells fixed at the indicated condition. Images of mAG1 (green) and Hoechst33342-stained nuclei (blue) were merged. Scale bars, 50 μm. (**c**) Western blot analysis of cells obtained in the same condition as shown in (**a**). Full-length blots are presented in Supplementary Figures 5.

There are several STAT3 inactivation mechanisms involved in dissociation of STAT3 dimerization. Suppressor of cytokine signaling (SOCS) family proteins or SHP2 phosphatase negatively regulate activation of STAT3 by JAK inhibition or dephosphorylation of phosphorylated STAT3^34–36^. To determine whether tyrosine dephosphorylation mechanisms occurred with the STAT3 fusion proteins, we observed puncta formation upon OSM stimulation and after OSM washout. The punctate signal increased and peaked 1 h after OSM washout, and gradually decreased to basal levels by 8 h after washout (Fig. 3a,b). Western blot analysis showed a time-dependent decrease of Y705 phosphorylation of endogenous STAT3 and PB1-mAG1-STAT3. Phosphorylation of endogenous STAT3 Y705 almost disappeared by 1 h after OSM washout, but it took more than 8 h for phosphorylation of exogenous STAT3 Y705 to disappear (Fig. 3c). These results indicate that the tyrosine dephosphorylation mechanisms occurred for both endogenous STAT3 and PB1-mAG1-STAT3. Thus, PB1-mAG1-STAT3 can be utilized to analyze the dynamic association and dissociation of the STAT3 homodimer reversibly in living cells.

#### Analysis of disease-related STAT3 somatic mutants using homoFluoppi

**Figure 4:**
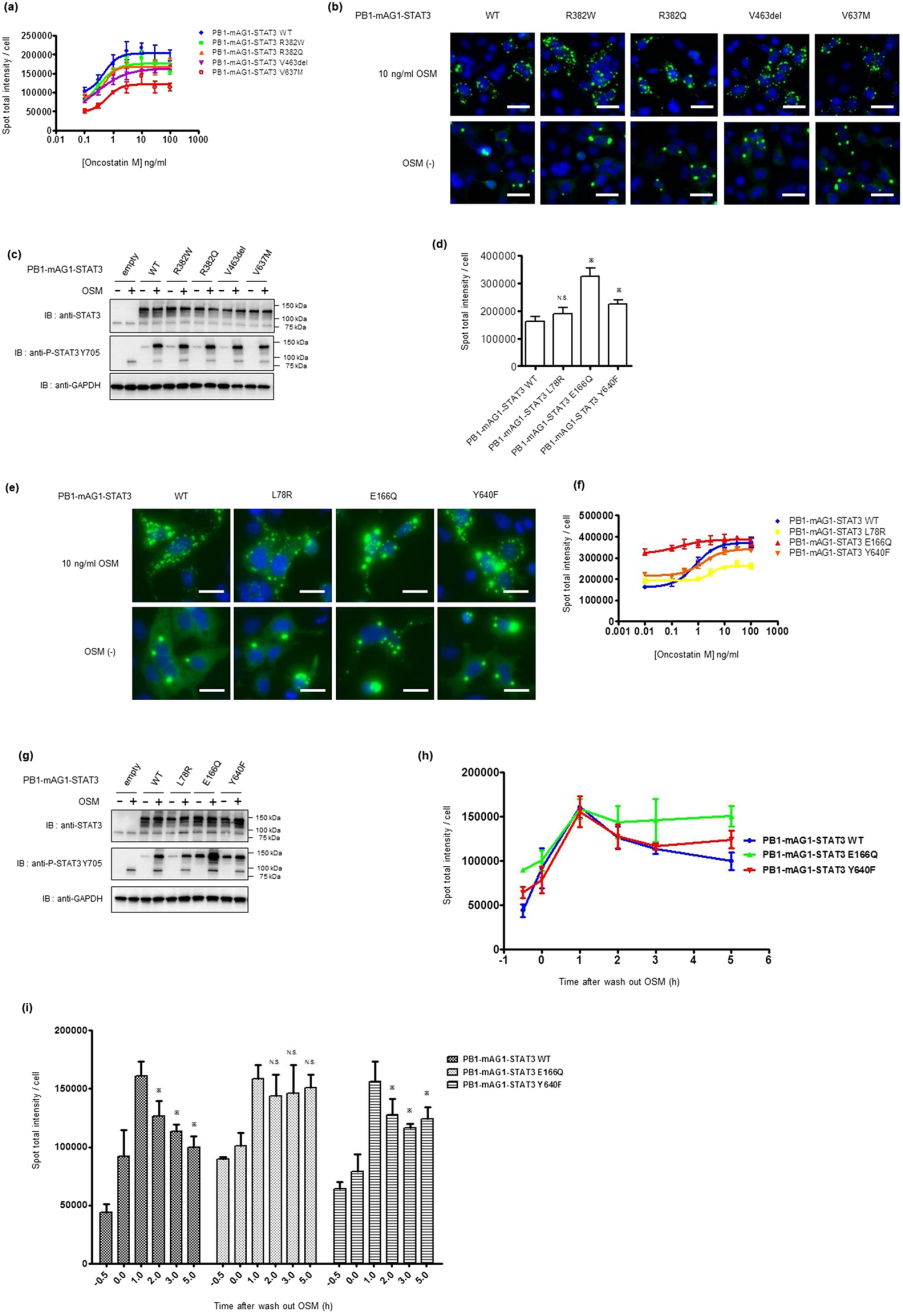
Analysis of disease-related STAT3 somatic mutants using homoFluoppi. (**a**) Dose (OSM)-response (fluorescent punctate intensity per cell) curve for cells expressing the indicated STAT3 WT or HIES mutant homoFluoppi constructs. OSM treatment was for 1 h. Each data point represents the mean of four replicates, and the error bars represent the standard deviation from the mean. (**b**) Representative fluorescent images of cells harvested at the indicated condition. Images of mAG1 (green) and Hoechst33342-stained nuclei (blue) were merged. Scale bars, 50 μm. (**c**) Western blot analysis of cells transiently express indicated STAT3 homoFluoppi constructs. OSM treatment was for 30 min. Full-length blots are presented in Supplementary Figures 5. (**d**) Fluorescent punctate intensity of cells expressing indicated homoFluoppi constructs without OSM. Each data point represents the mean of four replicates, and the error bars represent the standard deviation from the mean. Statistical significance was examined in the Student’s *t*-test with Bonferroni correction (*, p < 0.05 versus WT; N.S., not significant). (**e**) Fluorescent images of cells expressing indicated homoFluoppi construct with (top) or without (bottom) OSM. Images of mAG1 (green) and Hoechst33342-stained nuclei (blue) were merged. Scale bars, 25 μm. (**f**) Dose (OSM)-response (fluorescent punctate intensity per cell) curve for cells expressing indicated STAT3 WT or IHCA mutant homoFluoppi constructs. OSM treatment was for 1 h. Each point represents the mean of four replicates, and the error bars represent the standard deviation from the mean. (**g**) Western blot analysis of cells transiently expressing the indicated STAT3 homoFluoppi constructs. OSM treatment was for 30 min. Full-length blots are presented in Supplementary Figures 5. (**h**), (**i**) Cells expressing PB1-mAG1-STAT3 WT, E166Q and Y640F were treated with 10 ng/mL OSM for 30 min, and then OSM was washed out and cells were incubated in medium without OSM for the indicated time. Each point represents the mean of six replicates, and the error bars represent the standard deviation from the mean. Statistical significance was examined in the Student’s *t-*test (*p < 0.05 versus 1 h of each construct; N.S., not significant).

Some STAT3 somatic mutations are known to cause disease^9^. Therefore, we used the homoFluoppi system to determine whether these mutations affected STAT3 dimer formation, caused changes in response to cytokine stimulation, or caused a pathological state. Hyper IgE syndrome (HIES) is an immune deficiency syndrome; one cause of this disease is a specific mutation in the STAT3 gene^37,38^. To date, a variety of familial STAT3 mutations have been reported^37,38^, which are located in a specific domain of STAT3. Mutations located in the DNA-binding and SH2 domains are often observed in patients with Hyper IgE syndrome, so these mutation sites are called hotspots^37^. Among the hotspot mutations, we selected STAT3 R382W, R382Q, V463del, and V637M mutants to analyze the effect of each mutation on punctate formation using homoFluoppi. All of the Hyper IgE STAT3 mutants tested in this study formed puncta in response to OSM stimulation; however, only the STAT3 V637M mutant located in the SH2 domain showed an impaired response compared with STAT3 WT (Fig. 4a,b). Western blot analysis of tyrosine phosphorylation of these mutants indicated comparable phosphorylation with STAT3 WT (Fig. 4c). STAT3 mutations in Hyper IgE syndrome are proposed to have a dominant negative phenotype indicative of a defect in STAT3 target gene activation^38^. To confirm that the dominant negative phenotype also existed in HEK293 cells, we tested the HIES mutants in a STAT3-dependent reporter assay. The STAT3 R382W, R382Q, V463del, and V637M mutants had less transactivation activity than the empty vector-transfected control, indicating that these mutants acted as dominant negative mutants of endogenous STAT3 WT, as previously suggested^38^ (Supplementary Fig. 3b). Next, we analyzed several somatic mutations (STAT3 L78R, E166Q, and Y640F) that have been found in patients with inflammatory hepatocellular adenomas (IHCAs)^14^ which are benign liver tumors. Rebouissou *et al*.^39^ first reported activated IL-6 signaling by gain-of-function gp130 mutations in IHCA. Subsequently, the authors reported that a small population of patients with IHCA harbor somatic mutations in STAT3^14^. All of the aforementioned STAT3 mutations in this study reportedly activate STAT3 in the absence of IL-6. For example, the Y640F mutant is constitutively phosphorylated on Y705, forms a homodimer without IL-6 stimulation, and is hypersensitive to IL-6. To analyze the consequence of STAT3 IHCA mutations by homoFluoppi, we transfected PB1-mAG1-STAT3 L78R, E166Q, and Y640F into HEK293 cells and examined puncta formation in the absence and presence of OSM stimulation. STAT3 E166Q and Y640F mutants formed more puncta than WT in the absence of OSM (Fig. 4d,e). Puncta were formed in an OSM dose-dependent manner upon transfection of L78R, E166Q, and Y640F mutants (Fig. 4f). Western blot analysis showed that E166Q and Y640F mutants were phosphorylated on Y705 without OSM stimulation (Fig. 4g). Thus, we concluded that homoFluoppi can be used to analyze how disease-related mutations affect dimer formation and posttranslational modifications. To evaluate the mechanisms underlying the initiation and propagation of IHCA by STAT3 somatic mutations, we determined how IHCA mutants (STAT3 E166Q and Y640F) affected the dimer dissociation process. We found that these mutants were activated in the absence of OSM, so we expected prolonged dimer formation of these IHCA mutants. To observe dimer dissociation of these mutants, we observed puncta formed by OSM stimulation and after OSM washout. We observed that puncta formed by STAT3 WT and Y640F upon OSM stimulation reached maximum levels by 1 h, and gradually decreased (Fig. 4h,i). However, puncta formation in cells bearing STAT3 E166Q was sustained for 5 h, indicating that the E166Q mutant affected the duration of STAT3 homodimerization (Fig. 4h,i).

#### Development of a HTS system to investigate compounds that inhibit STAT3 homodimerization in living cells

**Figure 5:**
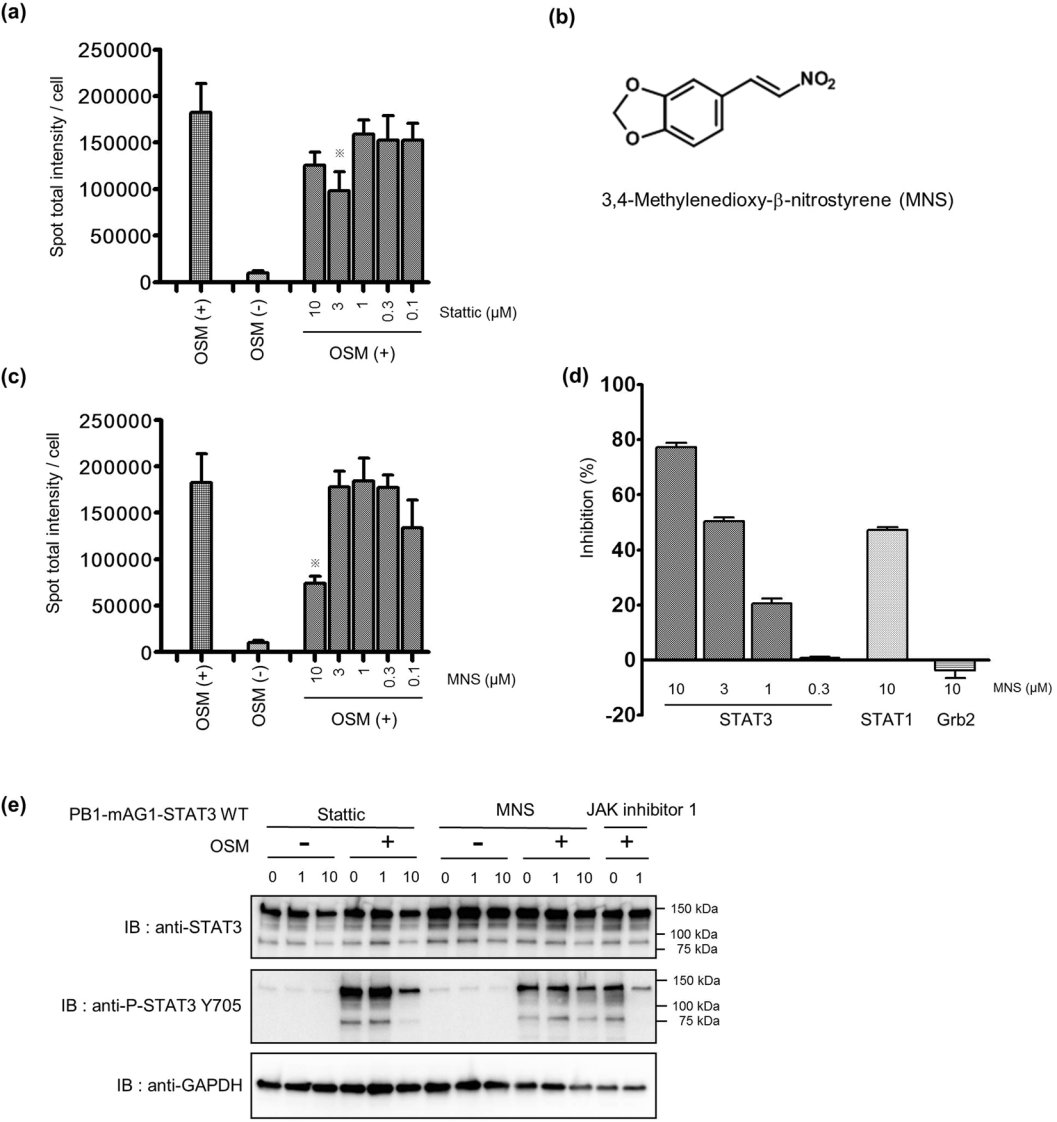
Development of HTS system to investigate compounds inhibiting STAT3 homodimerization in living cells. (**a**) Dose-dependent inhibition of STAT3 homoFluoppi by Stattic. Stattic treatment was 30 min before OSM stimulation. Cells were treated with 3 ng/mL OSM for 1 h. Each point represents the mean of four replicates, and the error bars represent the standard deviation from the mean. Statistical significance was examined in the Student’s *t*-test with Bonferroni correction (*, p < 0.05 versus OSM (+) with dimethyl sulfoxide (DMSO).; N.S., not significant). (**b**) Chemical structure of MNS (**c**). Dose-dependent inhibition of STAT3 homoFluoppi by MNS. MNS was added 30 min before treatment with 3 ng/mL OSM. Each point represents the mean of four replicates, and the error bars represent the standard deviation from the mean. Statistical significance was examined in the Student’s *t*-test with Bonferroni correction. (*p < 0.05 versus OSM (+) with DMSO; N.S., not significant). (**d**) Dose-dependent inhibition of MNS for STAT3 in the AlphaScreen, and selectivity for STAT1 and Grb2. Each point represents the mean of three replicates, and the error bars represent the standard deviation from the mean. (**e**) Western blot analysis of cells stably expressing PB1-mAG1-STAT3. Cells were treated with the indicated concentration of Stattic, MNS, and the JAK inhibitor 1, followed by treatment with or without 3 ng/mL OSM for 30 min. Full-length blots are presented in Supplementary Figures 5.

Activation of the STAT3 pathway causes several pathological conditions such as cancer^12^, and one of the important process underlying development of this pathological condition is dysregulation of STAT3 homodimerization^14^. Many reports have investigated the potential of using inhibitors of STAT3 homodimerization as a therapeutic strategy to modulate its activation in cancer^10^. However, most of these compounds were identified in cell-free screening systems due to the difficulty of observing homodimerization in cells for screening^40^. Therefore, we investigated whether the homoFluoppi system could be applied to high throughput screening (HTS) to identify compounds that inhibit STAT3 homodimerization in living cells. First, we calculated the Z’-factor of STAT3 homoFluoppi in 384-well plates to assess whether this assay system was compatible with the HTS method. The Z’-factor was above 0.4, indicating that this assay is applicable for HTS (Table 1). To confirm that the STAT3 inhibitor would show inhibitory effects in this assay conditions, we evaluated the effects of Stattic, a known inhibitor of STAT3 dimerization, which was discovered in a fluorescence polarization-based binding assay with the SH2 domain of STAT3^41^. We found that 3–10 μM Stattic partially inhibited punctate formation of STAT3 homoFluoppi (Fig. 5a). This suggested that we could use this system to find other compounds that inhibit STAT3 homodimerization. We performed HTS of a library of 461 compounds with activity toward known targets, and identified dozens of hit compounds, some of which were known to inhibit JAK. In addition to these known JAK inhibitors, we identified 3,4-methylenedioxy-β-nitrostyrene (MNS) as a STAT3 homodimerization inhibitor for the first time (Fig. 5b,c). To analyze the mechanisms underlying the decrease in STAT3 homodimerization by MNS in living cells, we examined MNS activity using the AlphaScreen assay, which measures the interaction of the STAT3 SH2 domain and phosphopeptide fused to donor and acceptor beads. When the interaction between the STAT3 SH2 domain and phosphopeptide occurs, a luminescent signal is emitted from the acceptor beads. The results showed that MNS had inhibitory effects in the AlphaScreen assay (Fig. 5d). To test the selectivity of this inhibitor, we examined the inhibitory effects of MNS toward Grb2 and STAT1, which are signal transduction proteins that also have an SH2 domain. MNS did not show inhibitory effects toward Grb2 and showed weak inhibitory effects toward STAT1. Previous reports have shown that MNS inhibits Syk and Src kinases^42,43^. Thus, we analyzed whether MNS influenced the phosphorylation of STAT3 Y705, and found no effects up to 10 μM MNS (Fig. 5e). We also confirmed that 10 μM Stattic reduced the phosphorylation of STAT3 Y705 as previously reported (Fig. 5e)^41^.

**Table 1:** Z’-factor and IC_50_ values of JAK inhibitor 1.

	1	2	3
Z’-factor	0.59	0.46	0.72
IC_50_ (nM)	6.0	13.7	27.4

These data were taken from three independent experiments. The Z’-factor was calculated from 32 control (with 3 ng/mL OSM) and 32 blank (without OSM) samples.

## Discussion

In this study, we validated that the homoFluoppi is a valuable research tool for observing the dynamic association and dissociation of STAT3 in living cells. We confirmed that phosphorylation of Y705 and an intact SH2 domain facilitate puncta formation. We also confirmed that we could observe both the association and dissociation processes of STAT3 dimerization reversibly using this technology. To this end we developed four STAT3 fusion constructs and selected the best one for detection of protein dimerization with the homoFluoppi system. In the case of STAT3 homodimerization, we determined that the PB1-mAG1 tag fused to the N-terminus of STAT3 was the best construct. Fluoppi tag (PB1 and mAG1) fusion to the C-terminus of STAT3 tended to be phosphorylated, because STAT3-PB1-mAG1 and STAT3-mAG1-PB1 were slightly phosphorylated on Y705 without OSM stimulation (Fig. 1b). Cells expressing STAT3-PB1-mAG1 and STAT3-mAG1-PB1 formed many puncta without OSM stimulation (Fig. 1d). In addition, apart from the effect of phosphorylation without OSM, the position of the tags may result in formation of numerous puncta of STAT3-PB1-mAG1 and STAT3-mAG1-PB1. FRET analysis of unphosphorylated STAT3^19^, showed that unphosphorylated STAT3 seemed to form a parallel dimer using SH2 domain interaction. As a result, the C terminus of each unphosphorylated STAT3 is placed closer than the N terminus region, which is similar to phosphorylated STAT3 dimer^30^. With regard to the detection mechanism of homoFluoppi, tagging the C-terminus of STAT3 might cause undesirable puncta formation. Therefore it was suggested that the proper position of homoFluoppi tags are required for the detection of both latent and activated STAT3 dimers.

To calculate punctate images, three approaches are available in ArrayScan Spot Detector BioApplication. In all of the three approaches, the PB1-mAG1-STAT3 construct afforded the increased signal intensity in an OSM concentration-dependent manner as shown in Fig. 1c (spot total intensity), Supplement Fig. 1a (spot total area) and Supplement Fig. 1b (spot total counts). Among them, the spot total intensity (Fig. 1c) was thought to be the best way to quantify the degree of STAT3 dimer formation. In spite of various three-dimensional shapes (sizes) of the fluorescent puncta, the images taken by ArrayScan are two-dimensional. This suggests that the real punctate volume would not always be consistent with spot total area or the spot total counts. Therefore we used spot total intensity to evaluate the degree of STAT3 homodimer.

Although PB1-mAG1-STAT3 was the optimal construct for detection of dynamic STAT3 dimerization in living cells, it did not increase transactivation activity, even in the presence of OSM (Supplementary Fig. 2b). The other homoFluoppi constructs tested also did not have transactivation activity (Supplementary Fig. 2b). This suggests that STAT3 homoFluoppi cannot be used to study the mechanisms underlying nuclear localization or chromatin binding of STAT3. The N-terminal coiled-coil domain of STAT3 is important for its nuclear localization^44^. The PB1-mAG1 tag fused to the N-terminus of STAT3 may inhibit interaction of the coiled-coil domain with essential molecules such as importin, which facilitates translocation to the nucleus^45–48^.

As shown in Fig. 3a–c, the punctate signal was gradually reduced in association with dephosphorylation of Y705 in PB1-mAG1-STAT3 after washing out OSM. Those results suggest that Tyr-phosphorylated PB1-mAG1-STAT3 could be a substrate for ubiquitously expressed Tyr-phosphatases such as SHP2^49^. The SOCS family proteins are known to be induced by the STAT3 transactivation as a negative feedback regulation. Therefore, the inactivation by SOCS seems not be involved in STAT3 homoFluoppi, because PB1-mAG1-STAT3 does not translocate to the nucleus (Supplementary Fig. 2a,b). This might be important feature of the STAT3 homoFluoppi system which enables us to analyse only cytoplasmic reaction.

 We demonstrated that R609Q mutation could neither form the punctate nor induce Tyr-phosphorylation of PB1-mAG1-STAT3 (Fig. 2b–d). It is well known that the SH2 domain is required for STAT protein binding to cytokine receptors and subsequent Tyr-phosphorylation by JAKs. An arginine residue conserved in all known SH2 domains recognizes phosphotyrosine^50^. Therefore, R609 in STAT3 is such a key residue for STAT3 activation and our results using STAT3 homoFluoppi were consistent with previous studies^18^. In addition, the PB1-mAG1 STAT3 R609Q mutant showed a significantly less punctate signal than WT (Fig. 2a) without OSM stimulation, indicating that homoFluoppi can be used to detect not only cytokine-dependent dimerization but also latent STAT3 dimerization in a similar manner as IP^31^, BRET^20^, and FRET^18,19^. The unique functional importance of latent STAT3 dimerization has been shown^51,52^, therefore, it is important to use a method that is sensitive enough to detect latent STAT3 dimerization in living cells, as was the case with the homoFluoppi system.

It is important to investigate how specific mutations found in patients affect signal transduction, in order to understand the underlying mechanisms and subsequently identify effective treatments. It is also important to investigate whether drugs have the desired effect on the mutated protein when considering whether a drug will be effective for different patient groups. In this study, we used homoFluoppi to examine how STAT3 somatic mutations reported in HIES and IHCAs affect the dynamics of STAT3 homodimer formation. In analyzing the HIES mutations, most of them showed comparable punctate formation as WT, so homodimer formation of these STAT3 mutants appeared normal. However, the STAT3 V637M mutant showed only slightly less punctate formation than STAT3 WT, and phosphorylation on Y705 of STAT3 V637M was comparable with WT in our experimental condition using HEK293 cells. Previous works have suggested decreased phosphorylation on Y705 in STAT3 V637M and other SH2 mutants of HIES (S611N, F621V) and loss of homodimerization in STAT3 S611N and F621V^53–55^. It is possible that the detection of STAT3 homodimers in our study differs from detection by IP assays, as homoFluoppi is a quantitative method. In the analysis of IHCA mutations, previous studies have suggested that STAT3 Y640F homodimerize independent of stimulus^14^. We also confirmed that STAT3 E166Q and Y640F homodimerized in the absence of OSM and activated reporter gene (Fig. 4c,d, Supplementary Fig. 3c). In addition, STAT3 E166Q caused prolonged dimer formation (Fig. 4h,i). Because STAT3 E166Q showed OSM-independent dimer formation, a breakdown of the balance between association and dissociation is suggested. Our results also suggest that one cause of IHCA by STAT3 mutations may be the extended dimerization time, which should be transient in normal signal transduction. Thus, the homoFluoppi system can be used to analyze somatic mutations present in signal transduction molecules that are related to disease initiation.

We developed a screening system to screen for inhibitors of STAT3 homodimerization in living cells using homoFluoppi, and subsequently identified MNS as a STAT3 inhibitor. MNS selectively inhibited STAT3 dimerization in the AlphaScreen assay compared to other SH2 domain-containing proteins (STAT1 and Grb2). Thus, MNS seemed to interact with the STAT3 SH2 domain and have selective inhibitory activity toward STAT3 in the AlphaScreen (Fig. 5d). However, MNS did not inhibit STAT3 phosphorylation of Y705 (Fig. 5e), so it appears to have a different inhibitory mechanism of STAT3 dimerization than Stattic^41^. He *et al*.^56^ reported that MNS inhibits NLRP3 inflammasome activation by directly inhibiting NLRP3, and that its inhibitory activity is independent of syk kinase inhibition. STAT3 is reported as an important player in the NLRP3 inflammasome^57^. Thus, the inhibitory effect of MNS towards STAT3 may involve inhibition of the NLRP3 inflammasome.

The homoFluoppi screening system can also be applied to mutated STAT3. For example, it may be ideal to find compounds that specifically inhibit the STAT3 protein with somatic undesired mutations. In the same way as was done with the STAT3 homodimer, many homodimers can be analyzed by homoFluoppi in living cells, as well as the mechanisms and posttranscriptional modifications underlying homodimer association and dissociation. Thus, homoFluoppi can be used as a research tool for investigating whether specific mutations found in patients affect homodimerization of that molecule. In addition, homoFluoppi can be used to screen potential candidates for drug development.

## Materials and Methods

### Cell culture and transfection

Human embryonic kidney (HEK) 293 cells were grown in the Dulbecco’s modified Eagle medium (DMEM) (Thermo Fisher Scientific) supplemented with 10% FBS. The cells were incubated at 37 °C in a humidified atmosphere containing 5% CO2. Transfection was done with Lipofectamine3000 (Invitrogen). Conditions and protocols were determined according to the manufacturer’s instructions. To establish cell line stably expressing PB1-mAG1-STAT3, transfected cells were cultured for 2 weeks in selection medium containing 500 μg/mL of Geneticin (Gibco). Single cell cloning was done by limiting dilution method. Each single clone was evaluated in the STAT3 homofluoppi system whether they respond to the stimulation.

### Western blotting

Total cell lysate were prepared using RIPA buffer (Wako) supplemented with protease inhibitor cocktail (Roche Diagnostics). Then total cell lysate were mixed with sample buffer (Nacalai Tesque) and boiled at 95 °C for 5 min. Samples were then separated on a 4–20% Mini-PROTEAN TGX Gel (Bio-rad) at 200 V for 35 min. The separated proteins were transferred to a PVDF membrane using an iBlot system (Invitrogen). The membrane was blocked with PVDF Blocking Reagent for Can Get Signal (TOYOBO). The membrane was incubated with primary antibody. Antibody toward Phospho tyrosine 705 of STAT3 (#4113, Cell Signaling Technology, 1:2000), STAT3 (#12640; Cell Signaling Technology, 1:1000) and GAPDH (#2118; Cell Signaling Technology, 1:1000) were used. Horseradish peroxidase (HRP) conjugated anti-rabbit IgG (Thermo Fisher Scientific) and anti-mouse IgG (SouthernBiotech) were used as a secondary antibody. Detection was carried out with SuperSignal West Femto Maximum Sensitivity substrate (Thermo scientific). Visualization of images was performed by a LAS1000 imaging system (GE Healthcare life sciences).

### AlphaScreen

AlphaScreen STAT3, STAT1 or Grb2 binding assays were performed as described in the previous report^58^. Briefly, these assays were carried out in the buffer containing 10 mM HEPES-NaOH (pH 7.4), 50 mM NaCl, 1 mM EDTA (pH 8.0), 0.1% (w/v) NP-40, and 10 ng/mL (w/v) BSA with 96 well microplate (Coster 3693). Phospho-Tyr (pTyr) peptide probes used in this study were 5-carboxyfluorescein (FITC)-GpYLPQTV for STAT3, FITC-GpYDKPHVL for STAT1, and FITC-PSpYVNVQN for Grb2. Each SH2-containing biotinylated protein (75 nM of STAT3, 30 nM of STAT1 or 6 nM Grb2) was incubated with a test compound for 15 min. Each protein sample was then incubated for 90 min with its corresponding FITC-pTyr peptide (0.05 nM for STAT3, 0.72 nM for STAT1 or 0.18 nM for Grb2), and mixed with streptavidin-coated donor beads and anti-FITC acceptor beads simultaneously before detection at 570 nm using EnVison Xcite (PerkinElmer).

### Construction of plasmids

Full length human STAT3 (NM_139276) were cloned to homoFluoppi vector. Mutant STAT3 vectors were constructed using KOD -Plus- Mutagenesis Kit (TOYOBO, Japan) and primers listed in Table S1. All vectors were verified by sequencing.

### STAT3 dimerization assay

Cells transiently or stably express homoFluoppi tag fused STAT3 were seeded in 384-well plate coated with poly D lysine (Corning) using DMEM supplemented with 0.5% (v/v) Fetal bovine serum and cultured for 24 hours to make cells quiescent state. The cells were treated with OSM (Sigma-Aldrich), IL-6 (R&D systems) or IFN alpha (Sigma-Aldrich) for indicated time. For screening of dimerization inhibitor, each compound was applied 30 min before 3 ng/ml OSM stimulation. Then the cells were fixed with 4% paraformaldehyde and stained with 1ng/ml Hoechst 33342. Imaging and Image analysis of the cells were automatically proceeded using an ArrayScan XTI HCS Reader (Thermo Fisher Scientific). Filter set for the blue fluorescent (Hoechst 33342–labeled nuclei) and green fluorescent (PB1-mAG1-STAT3) were used. Images were obtained for 9 fields per well. Spot Detector BioApplication was used for the analysis after optimising manufacture’s settings. In Spot Detector BioApplication, Hoechst-labeled nuclear images were used to identify individual cells and region of interest (ROI) to find puncta. Green fluorescent puncta were identified, then total spot fluorescent intensities, spot area and number of spot within the images were analyzed automatically. To compare the different well, the total spot fluorescent intensity, spot area and number of spot was divided by the number of fluorescent cells in the images. Spot fluorescent intensities per each cell were used as an index of dimer formation and applied for the calculation of inhibitory effect of test compounds.

In compound screening, signals for the wells treated with OSM and DMSO were represented as 0% and 100% inhibition, respectively. The IC_50_ value was calculated by Graph pad prism 5.0. The Z’ factor was calculated using the equation Z’ = 1–(3xSD_OSM + 3xSD_DMSO)/(Signal_OSM - Signal_DMSO).

### Reporter gene assay

Cignal Reporter Assay Kits (Qiagen) and Dual-Glo™ Luciferase Assay System (promega) were used to evaluate STAT3 dependent transactivation activity. STAT3 expression vectors or control vectors were transfected to HEK 293 cells with reporter plasmids. 24 hours after transfection, cells were plated on 384-well plate using DMEM supplemented with 0.5% (v/v) fetal bovine serum. 24 hours after plating, cells were treated with OSM for 4 hours. Then, the Dual-Glo® Luciferase Reagent was applied to each well and incubated for 20 min at room temperature. The luciferase signals from firefly luciferase were detected by EnVision (Perkinekmer). After 5 min, Dual-Glo® Stop & Glo® Reagent was added to each well and incubated for 20 min at room temperature. The luciferase signals from Renilla luciferase were detected by EnVision (Perkinekmer). The relative signal intensity was calculated by dividing reporter signal

The datasets generated during and/or analysed during the current study are available from the corresponding author on reasonable request.

## Electronic supplementary material

Supplementary figures
