# Supplementary material for: Visualization and quantification of dynamic STAT3 homodimerization in living cells using homoFluoppi
Source: Sci Rep. 2018 Feb 5;8:2385. doi: 10.1038/s41598-018-20234-2 (PMC5799161; doi:10.1038/s41598-018-20234-2)
Supplement: Supplementary file 1 — Supplementary figures [file 41598_2018_20234_MOESM1_ESM.pdf]

Visualization and quantification of dynamic STAT3 Homo-dimerization in living cells using homoFluoppi.

Author list (Yusuke Okada, Taku Watanabe, Toru Shoji, Kyoko Taguchi, Naohisa Ogo, and Akira Asai)

Supplementary Figure. 1

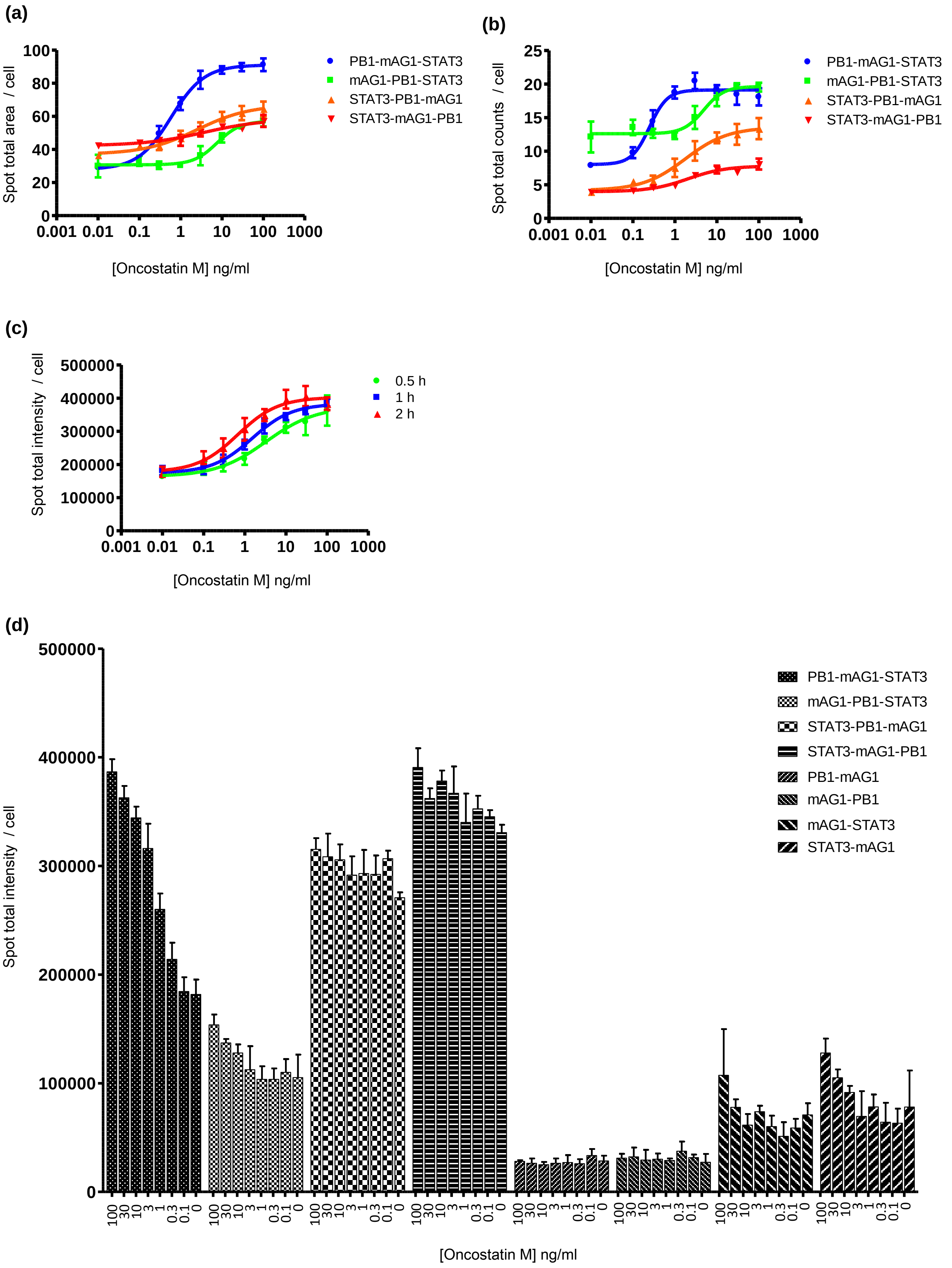

(e)

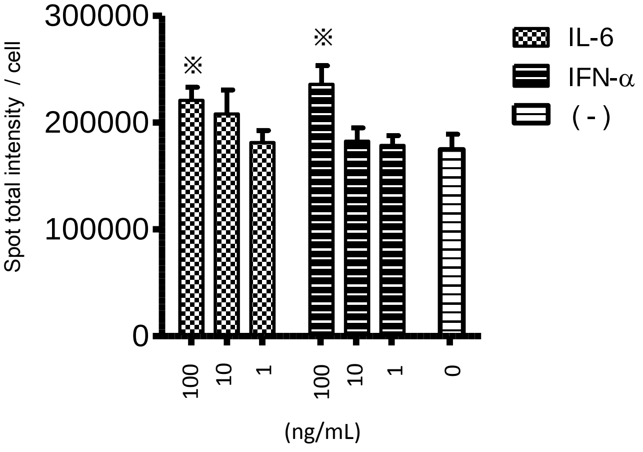

Supplementary Figure 1. Selection of the optimal construct for detection of STAT3 dimerization in living cells.

(a, b) Dose (OSM)-response (fluorescent punctate area (a) and counts (b) per cell) curve for cells expressing the indicated homoFluoppi constructs. Fluorescent punctate area and counts per cell was obtained by dividing the total mAG1 (green) fluorescent puncta area and counts by the total number of cell (nuclei), which had fluorescence in each field of view. Each point represents the mean of four replicates, and the error bars represent the standard deviation from the mean. (c) Dose (OSM)-response (fluorescent punctate intensity per cell) curve for cells expressing PB1-mAG1-STAT3. OSM were treated for indicated time. Fluorescent punctate intensity per cell was obtained by dividing the total mAG1 (green) fluorescent punctate intensity by the total number of cell (nuclei), which had fluorescence in each field of view. Each point represents the mean of four replicates, and the error bars represent the standard deviation from the mean. (d) Each bar represented fluorescent punctate intensity of cells expressing the indicated homoFluoppi construct. These cells were treated with indicated concentrations of OSM for 1 h. Each data point represents the mean of four replicates, and the error bars represent the standard deviation from the mean. (e) Fluorescent punctate intensity of cells expressing PB1-mAG1-STAT3 constructs treated with IL-6 (left) or IFN-α (right) for 1h. Data represent treatment with IL-6 or IFN-α from four replicates, and untreated cells represent the mean of eight replicates. The error bars represent the standard deviation from the mean. Statistical significance was examined using the Student's *t*-test with Bonferroni correction (*, $p<0.05$ versus untreated cells).

Supplementary Figure. 2

(a)

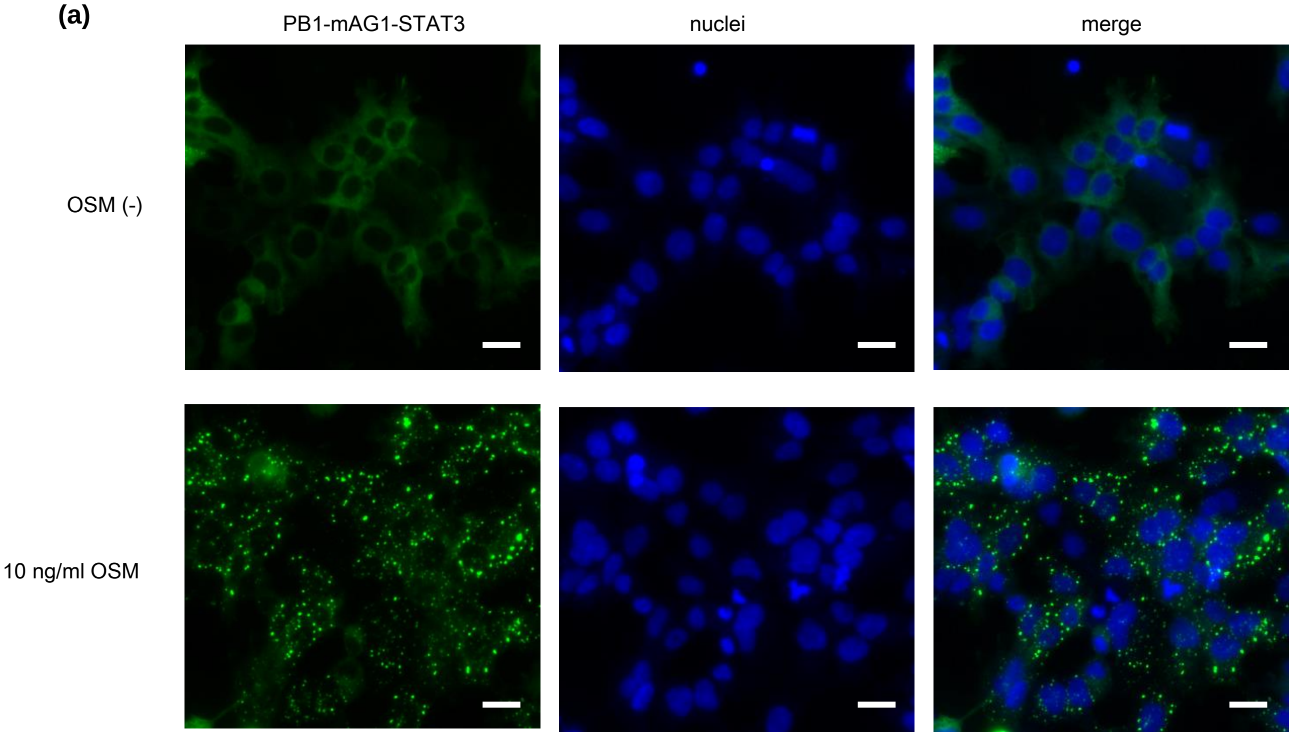

(b)

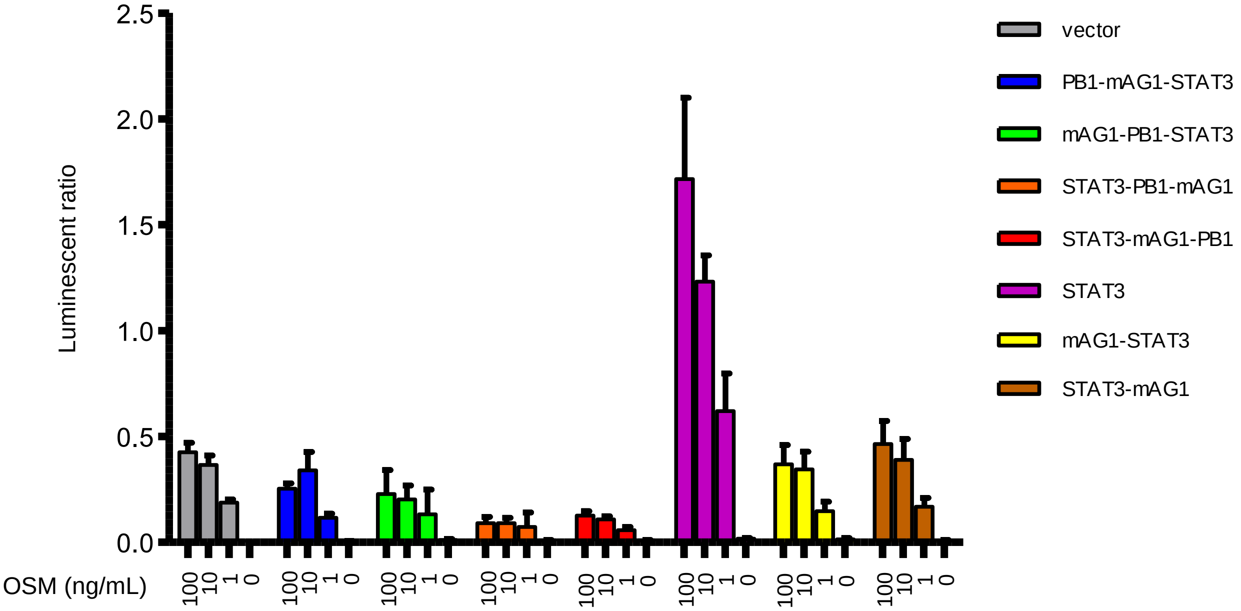

Supplementary Figure 2. Analysis of PB1-mAG1-STAT3 behavior in cells.
(a) Fluorescent images of cells expressing PB1-mAG1-STAT3 without (top) OSM or treated with (bottom) 10 ng/mL OSM for 1 h. Fluorescent images of PB1-mAG1-STAT3 (mAG1, left), Hoechst33342-stained nuclei (middle), and merged images (right) are shown. Scale bars, 25 µm. (b) Dose (OSM)-response (ratio of firefly luminescence signal divided by Renilla luminescence signal) data in the STAT3-dependent luciferase assay. Indicated concentrations of OSM were added and incubated for 4 h, and then the luminescent signals were read. Each point represents the mean of four replicates, and the error bars represent the standard deviation from the mean.

Supplementary Figure. 3

(a)

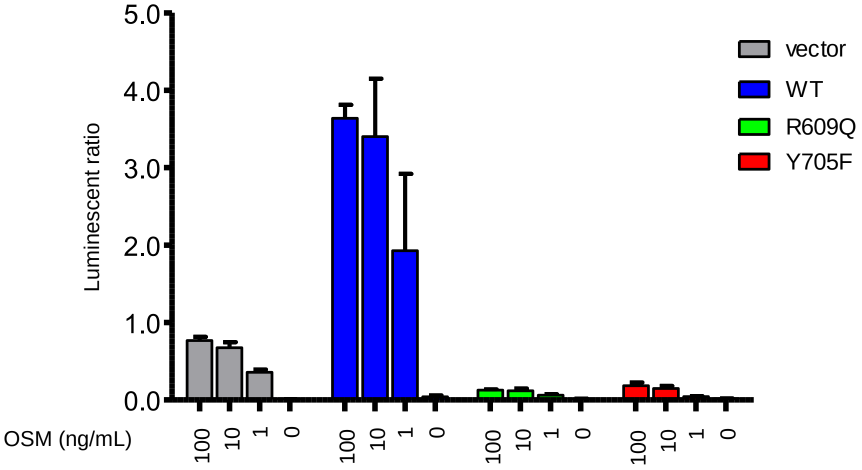

(b)

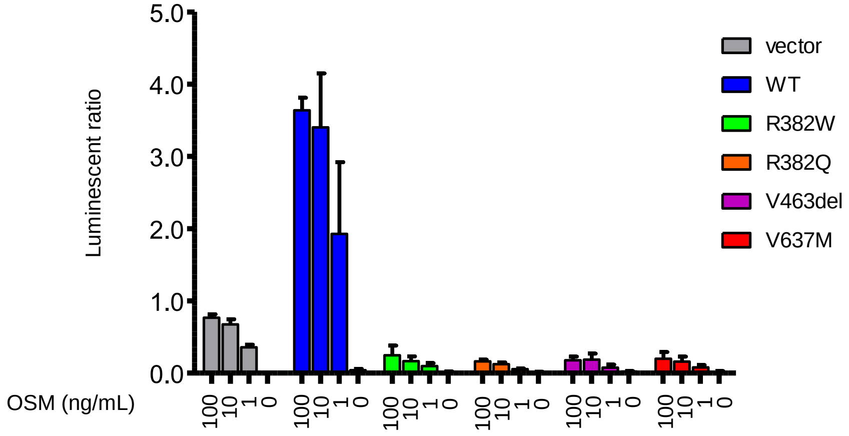

(c)

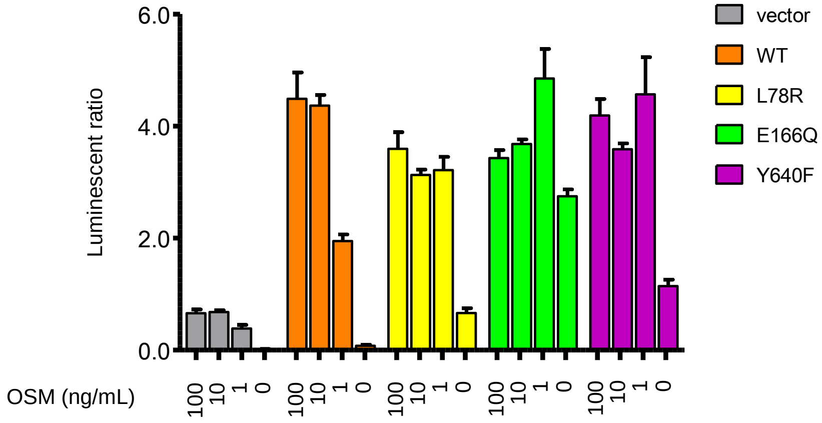

Supplementary Figure 3. Transactivation activity of the homoFluoppi tag removed STAT3 mutants.
(a-c) Transactivation activity of indicated homoFluoppi tag-removed STAT3 mutants. Dose (OSM)-response (ratio of firefly luminescence signal divided by Renilla luminescence signal) data of STAT3-dependent luciferase assay. Indicated concentrations of OSM were added and incubated for 4 h, and then the luminescent signals were. Each point represents the mean of four replicates, and the error bars represent the standard deviation from the mean.

Supplementary Figure. 4

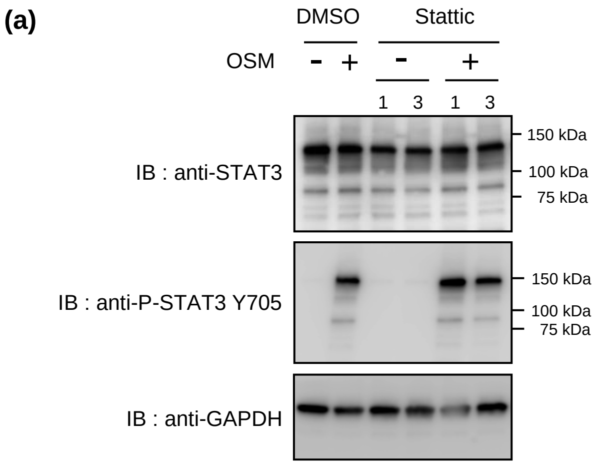

Supplementary Figure 4. Western blot analysis Stattic of STAT3 Y705 phosphorylation
(a) Western blot analysis of cells stably expressing PB1-mAG1-STAT3. Cells were treated with the indicated concentration of Stattic followed by treatment with or without 3 ng/mL OSM for 30 min. The full-length blots were cropped to improve clarity. Full-length blots are presented in Supplementary Figure 5.

Supplementary Figure. 5

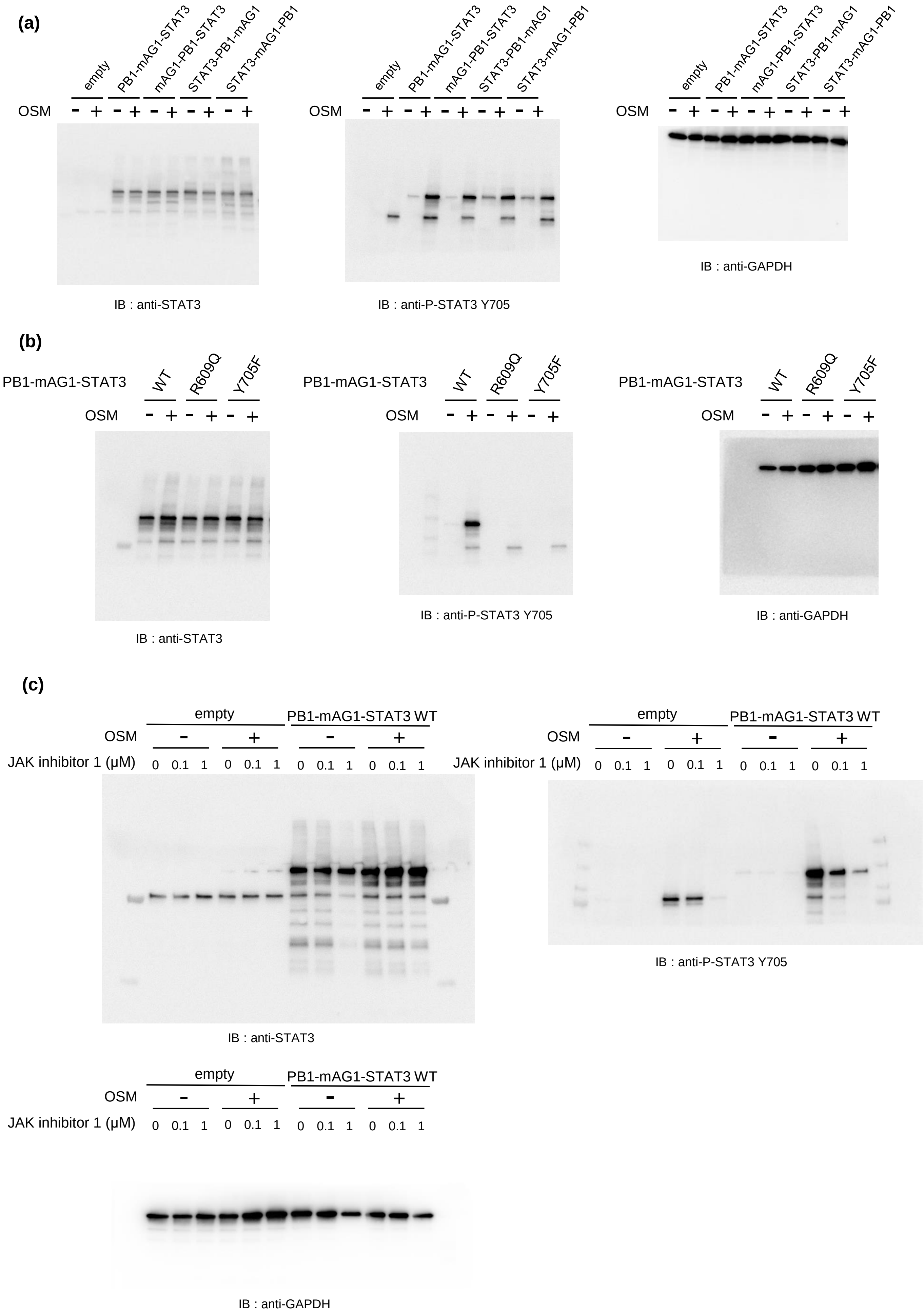

Supplementary Figure. 4 (continued)

(d)

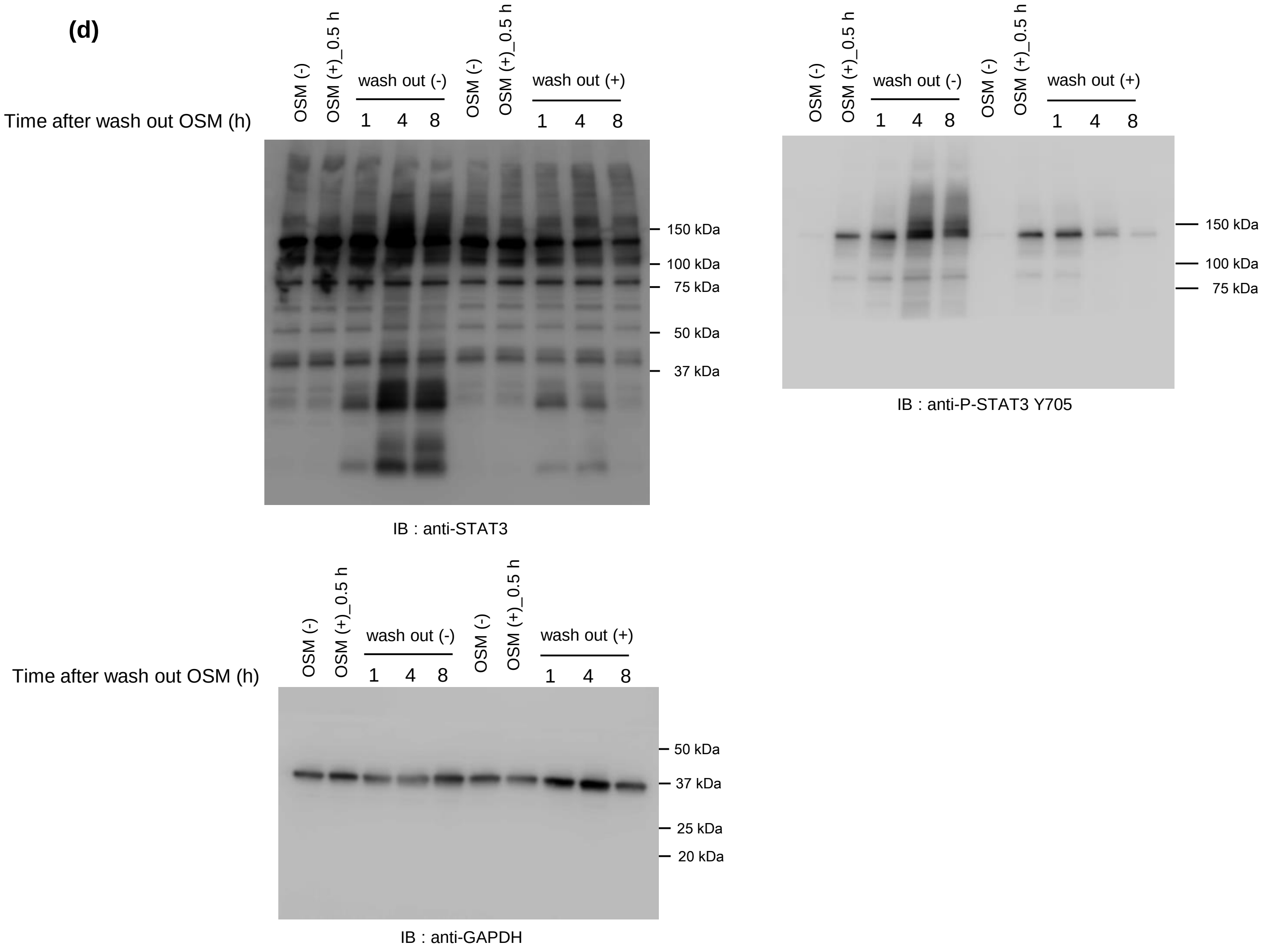

(e)

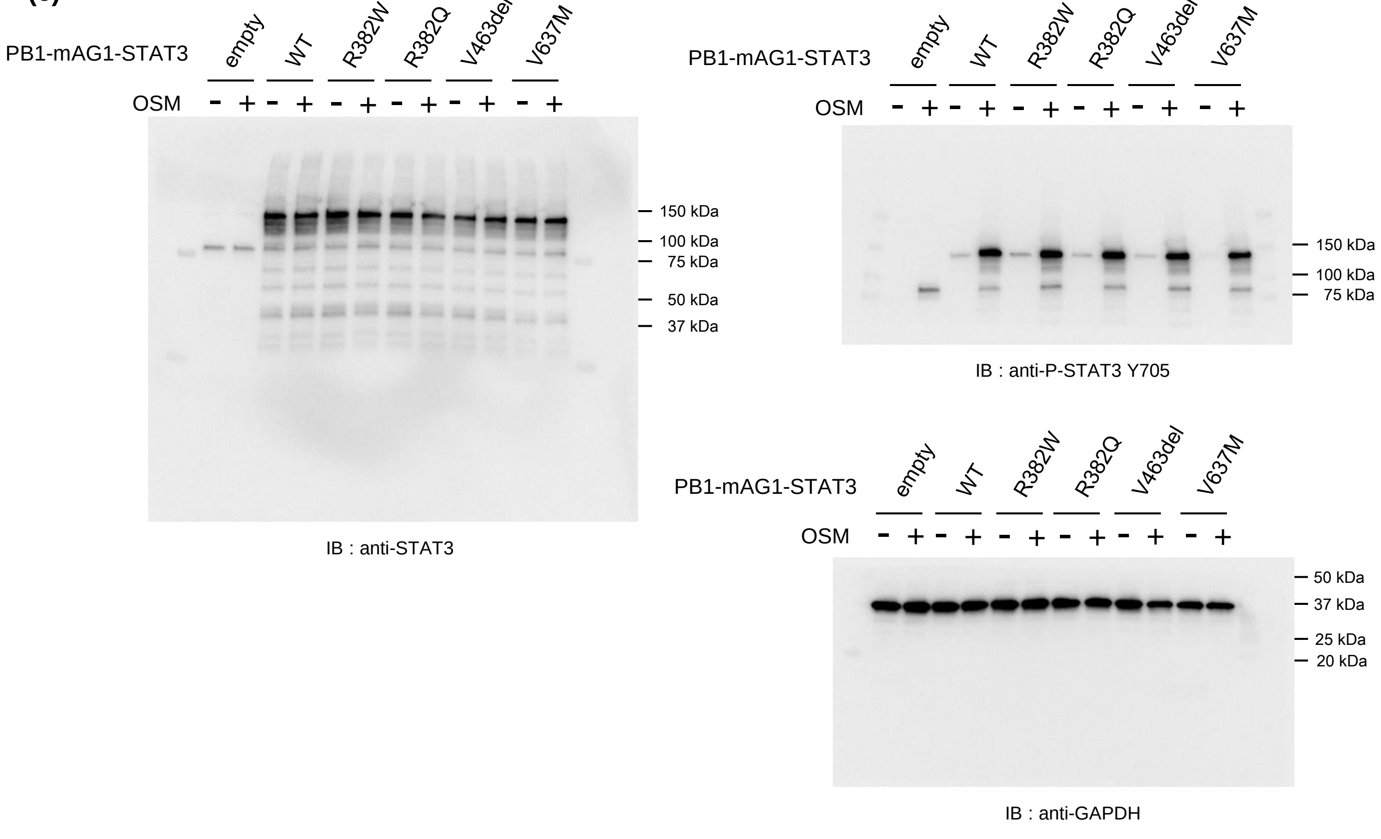

Supplementary Figure. 5 (continued)

(f)

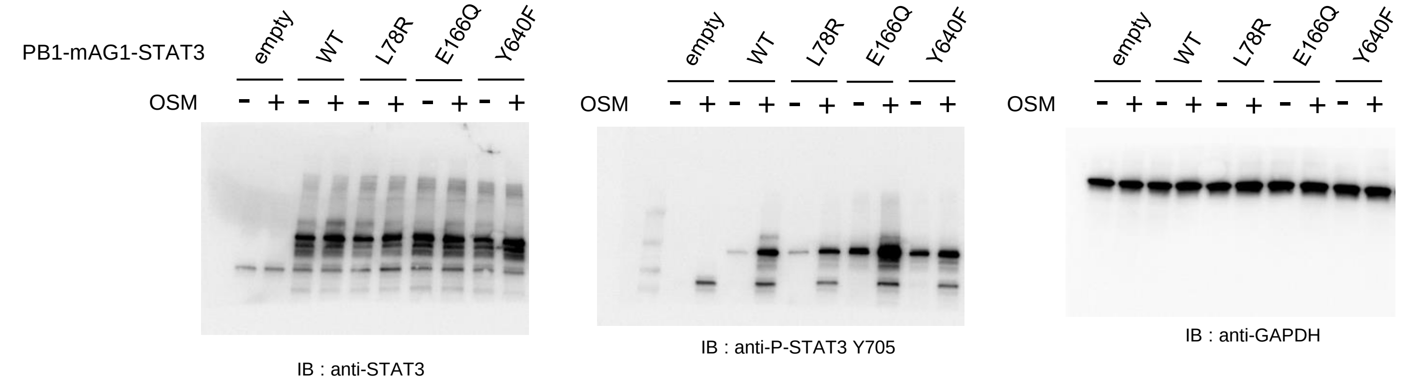

(g)

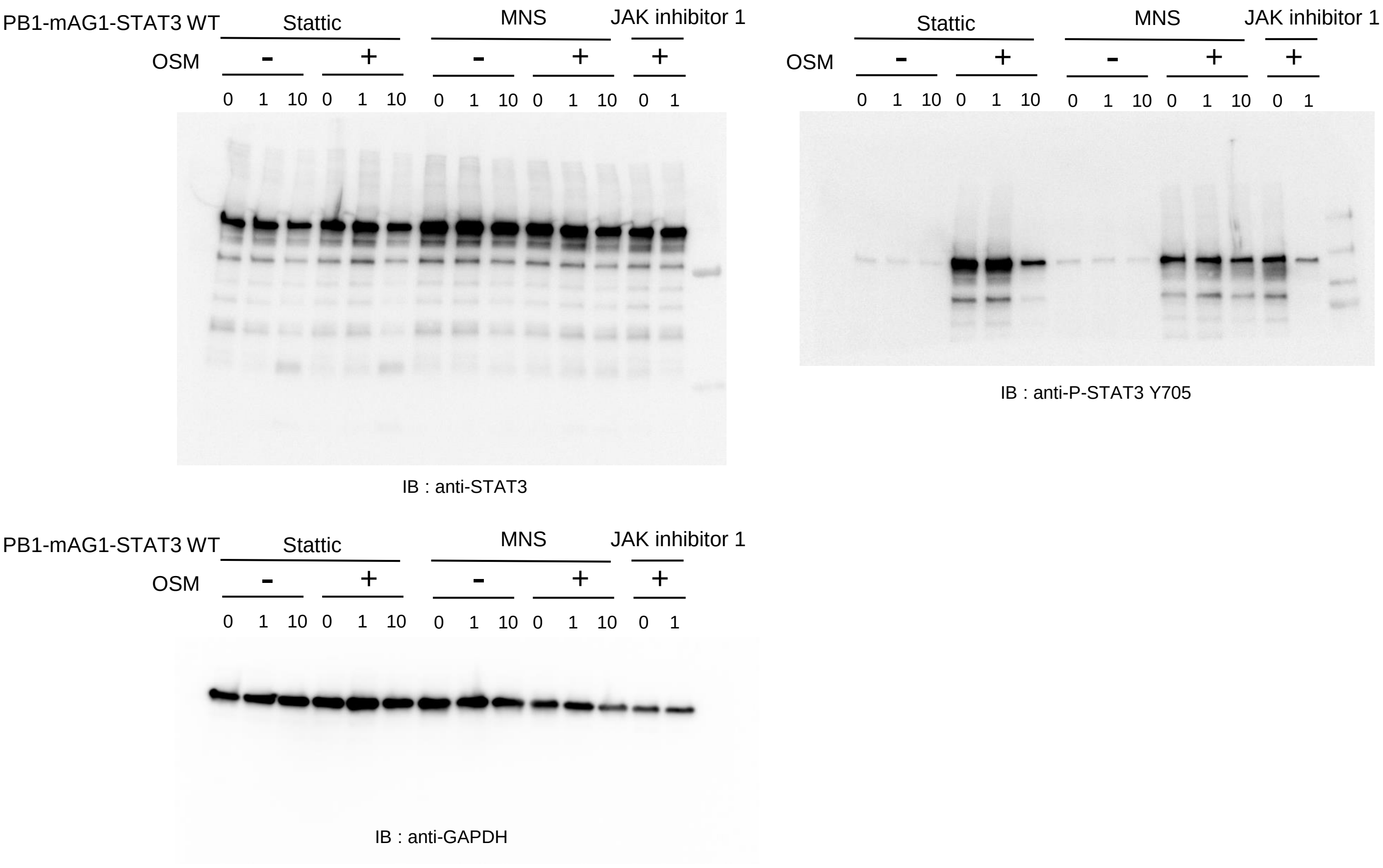

h

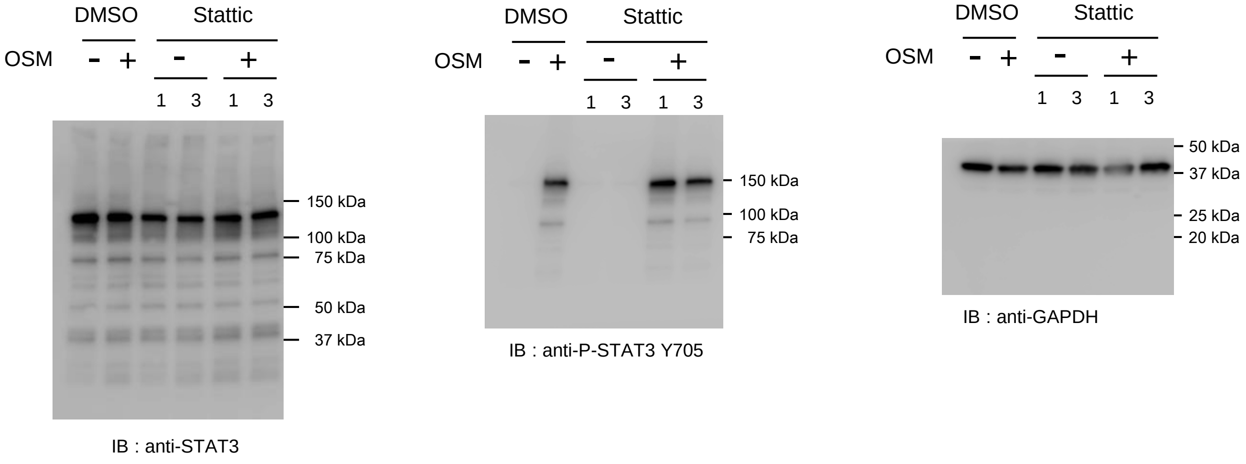

Supplementary Figure 5. Full-length blot data.
(a-h) Full-length blot data of Fig. 1b (a), Fig. 2d (b), Fig. 2g (c), Fig. 3c (d), Fig. 4c (e), Fig. 4g (f), Fig. 5e (g) and supplement Fig. 4a (h) were shown.

Supplementary Table. 1

Supplementary Table 1. Primer list.

NAME	Sequence (5‘ → 3')
STAT3-R609Q-Fw2	CAATTCAGTGAAAGCAGCAAAGAAGGA
STAT3-R609Q-Rv1	TAGCAGGAAGGTGCCTGGAGGCTTAGTG
STAT3-Y705F-Fw3	GCCCCATTCCTGAAGACCAAGTTTATCTGTG
STAT3-Y705F-RV3	AGCGCTACCTGGGTCAGCTTCAGGATG
STAT3-L78R-Fw1	GCTATCAGCACAATCTACGAAGAATC
STAT3-L78R-Rv1	GAACATTCGACTCTTGCAGGAAGCG
STAT3-E166Q-Fw1	CAGAATCTCCAGGATGACTTTGATTT
STAT3-E166Q-Rv1	TACCACTTTCATTTTCTGTTCTAG
STAT3-Y640F-Fw1	TCACAAAGCAGCAGCTGAACAACATG
STAT3-Y640F-Rv1	ATGGTTCCACGGACTGGATCTGGGTC
STAT3-R382W-Fw1	TGGAAATTTAACATTCTGGGCACAAAC
STAT3-R382W-Rv1	GGATCCTCTGAGAGCTGCAACGTC
STAT3-R382Q-Fw1	AGAAATTTAACATTCTGGGCACAAACAC
STAT3-R382Q-Rv1	GGGATCCTCTGAGAGCTGCAAC
STAT3-V463del-Fw1	ATCTCCAACATCTGTCAGATGCCAAATGC
STAT3-V463del-Rv1	CACAACTGGCAAGGAGTGGGTCTCTAG
STAT3-V637M-Fw1	ATGGAACCATACACAAAGCAGCAGCTG
STAT3-V637M-Rv1	GGACTGGATCTGGGTCTTACCGCTGATG
